# (2*Z*)-3-(3-Bromo­anilino)-1-(5-hy­droxy-3-methyl-1-phenyl-1*H*-pyrazol-4-yl)but-2-en-1-one

**DOI:** 10.1107/S1600536812006939

**Published:** 2012-02-24

**Authors:** Abdullah M. Asiri, Hassan M. Faidallah, Seik Weng Ng, Edward R. T. Tiekink

**Affiliations:** a Chemistry Department, Faculty of Science, King Abdulaziz University, PO Box 80203, Jeddah, Saudi Arabia; b The Center of Excellence for Advanced Materials Research, King Abdulaziz University, Jeddah, PO Box 80203, Saudi Arabia; c Department of Chemistry, University of Malaya, 50603 Kuala Lumpur, Malaysia

## Abstract

In the title compound, C_20_H_18_BrN_3_O_2_, the central carbonyl group forms amine-N—H⋯O and hy­droxy-O—H⋯O hydrogen bonds, which lead to two fused *S*(6) rings. The *N*-bound phenyl ring is coplanar with the five-membered ring to which it is attached [dihedral angle = 5.22 (18)°], but the dihedral angle [33.87 (17)°] between the terminal phenyl and bromo­benzene rings indicates an overall twist in the mol­ecule. In the crystal packing, mol­ecules assemble into dimeric aggregates *via* C—H⋯π inter­actions.

## Related literature

For background to the synthesis, see: Gelin *et al.* (1983 ▶); Bendaas *et al.* (1999 ▶). For the structures of the 4-chloro and 4-meth­oxy derivatives, see: Asiri, Al-Youbi, Alamry *et al.* (2011 ▶); Asiri, Al-Youbi, Faidallah *et al.* (2011 ▶).
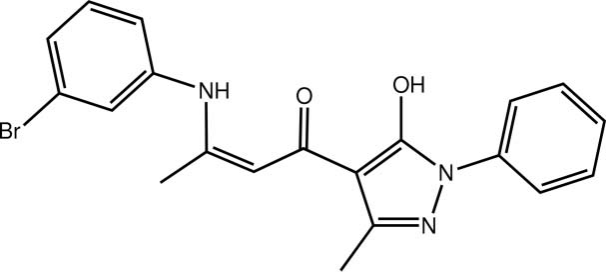


## Experimental

### 

#### Crystal data

C_20_H_18_BrN_3_O_2_
*M* *_r_* = 412.28
Monoclinic, 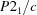

*a* = 8.7065 (5) Å
*b* = 11.7982 (8) Å
*c* = 17.5954 (12) Å
β = 101.536 (6)°
*V* = 1770.9 (2) Å^3^
*Z* = 4
Mo *K*α radiation
μ = 2.34 mm^−1^
*T* = 100 K
0.25 × 0.10 × 0.05 mm

#### Data collection

Agilent SuperNova Dual diffractometer with an Atlas detector
Absorption correction: multi-scan (*CrysAlis PRO*; Agilent, 2011 ▶) *T*_min_ = 0.592, *T*_max_ = 0.892
7812 measured reflections
4041 independent reflections
3033 reflections with *I* > 2σ(*I*)
*R*_int_ = 0.048

#### Refinement

*R*[*F* ^2^ > 2σ(*F* ^2^)] = 0.047
*wR*(*F* ^2^) = 0.105
*S* = 1.01
4041 reflections
245 parameters
2 restraints
H atoms treated by a mixture of independent and constrained refinement
Δρ_max_ = 0.58 e Å^−3^
Δρ_min_ = −0.47 e Å^−3^

### 

Data collection: *CrysAlis PRO* (Agilent, 2011 ▶); cell refinement: *CrysAlis PRO*; data reduction: *CrysAlis PRO*; program(s) used to solve structure: *SHELXS97* (Sheldrick, 2008 ▶); program(s) used to refine structure: *SHELXL97* (Sheldrick, 2008 ▶); molecular graphics: *ORTEP-3* (Farrugia, 1997 ▶) and *DIAMOND* (Brandenburg, 2006 ▶); software used to prepare material for publication: *publCIF* (Westrip, 2010 ▶).

## Supplementary Material

Crystal structure: contains datablock(s) global, I. DOI: 10.1107/S1600536812006939/sj5199sup1.cif

Structure factors: contains datablock(s) I. DOI: 10.1107/S1600536812006939/sj5199Isup2.hkl

Supplementary material file. DOI: 10.1107/S1600536812006939/sj5199Isup3.cml

Additional supplementary materials:  crystallographic information; 3D view; checkCIF report

## Figures and Tables

**Table 1:** Hydrogen-bond geometry (Å, °) *Cg*1 is the centroid of the N1/N2/C7–C9 ring.

*D*—H⋯*A*	*D*—H	H⋯*A*	*D*⋯*A*	*D*—H⋯*A*
O1—H1⋯O2	0.84 (1)	1.68 (3)	2.468 (3)	154 (6)
N3—H3⋯O2	0.88 (1)	1.87 (3)	2.617 (4)	142 (4)
C14—H14*B*⋯*Cg*1^i^	0.98	2.69	3.495 (4)	140

Symmetry code: (i) 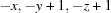.

**Fig. 1.**
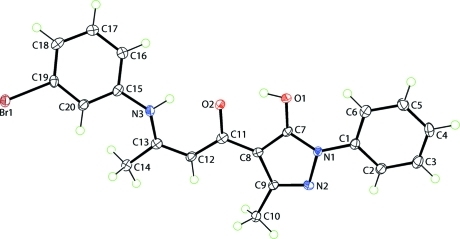
The molecular structure of (I) showing the atom-labelling scheme and displacement ellipsoids at the 50% probability level.

**Fig. 2.**
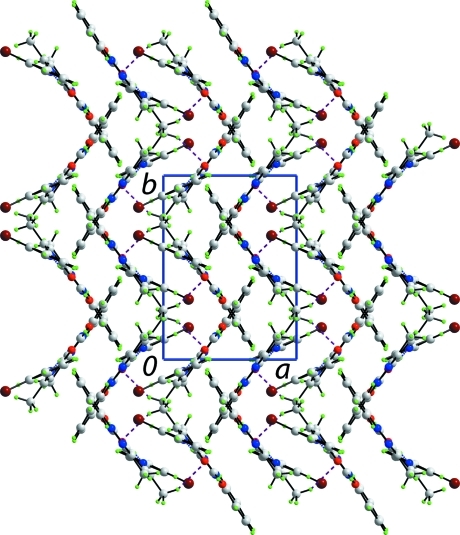
A view in projection down the c axis of the unit-cell contents of (I). The C—H···π interactions are shown as purple dashed lines.

## supplementary crystallographic information

### Comment

The title compound, 3-(3-bromoanilino)-1-(5-hydroxy-3-methyl-1-phenyl-1*H*-pyrazol-4-yl)but-2-en-1-one (I), was synthesized during investigations of reactions between pyrazoles and aniline derivatives based on literature precedents (Gelin *et al.*, 1983; Bendaas *et al.*, 1999) and was one of several compounds that were isolated in crystalline form (Asiri, Al-Youbi, Alamry *et al.*, 2011; Asiri, Al-Youbi, Faidallah *et al.*, 2011). As a continuation of these structural studies, the analysis of (I) is now described.

In (I), Fig. 1, The configuration about the formal C12═C13 bond [1.376 (4) Å] is *Z*. This arrangement allows the central O2-carbonyl atom to accept two hydrogen bonds from the adjacent hydroxyl and amine groups to close a pair of fused *S*(6) rings, Table 1. While the *N*-bound phenyl ring is co-planar with the five-membered ring to which it is connected, forming a dihedral angle of 5.22 (18)°, a twist in the molecule is evident as seen in the dihedral angle formed between the terminal phenyl and bromobenzene rings, dihedral angle = 33.87 (17)°.

The most notable feature of the crystal packing is the formation of C—H···π interactions where the π-system is the five-membered ring, Table 1. The resulting dimeric aggregates assemble into zigzag layers in the *bc* plane and stack along the *a* axis, Fig. 2.

### Experimental

A solution of 4-acetoacetyl-5-hydroxy-3-methyl-1-phenylpyrazole (0.005 mol) and 3-bromo-aniline (0.005 mol) in ethanol (25 ml) was refluxed for 2 h. The precipitate obtained from the hot solution was collected washed with methanol and recrystallized from its ethanol-benzene solution to provide yellow crystals; *M*.pt: 412–413 K.

### Refinement

Carbon-bound H-atoms were placed in calculated positions [C—H = 0.95 to 0.98 Å, *U*_iso_(H) = 1.2 to 1.5*U*_eq_(C)] and were included in the refinement in the riding model approximation. The N—H and O—H-atoms were located in a difference Fourier map, and were refined with distance restraints of N—H = 0.88±0.01 and O—H = 0.84±0.01 Å, respectively; their *U*_iso_ values were refined. Owing to poor agreement, the (0 0 2), (0 11 1), (1 11 2) and (1 9 4) reflections were omitted from the final cycles of refinement.

### Crystal data

**Table:**

C_20_H_18_BrN_3_O_2_	*F*(000) = 840
*M**_r_* = 412.28	*D*_x_ = 1.546 Mg m^−^^3^
Monoclinic, *P*2_1_/*c*	Mo *K*α radiation, λ = 0.71073 Å
Hall symbol: -P 2ybc	Cell parameters from 1846 reflections
*a* = 8.7065 (5) Å	θ = 2.4–27.5°
*b* = 11.7982 (8) Å	µ = 2.34 mm^−^^1^
*c* = 17.5954 (12) Å	*T* = 100 K
β = 101.536 (6)°	Bead, yellow
*V* = 1770.9 (2) Å^3^	0.25 × 0.10 × 0.05 mm
*Z* = 4	

### Data collection

**Table:**

Agilent SuperNova Dual diffractometer with an Atlas detector	4041 independent reflections
Radiation source: SuperNova (Mo) X-ray Source	3033 reflections with *I* > 2σ(*I*)
Mirror monochromator	*R*_int_ = 0.048
Detector resolution: 10.4041 pixels mm^-1^	θ_max_ = 27.6°, θ_min_ = 2.4°
ω scan	*h* = −10→11
Absorption correction: multi-scan (*CrysAlis PRO*; Agilent, 2011)	*k* = −11→14
*T*_min_ = 0.592, *T*_max_ = 0.892	*l* = −14→22
7812 measured reflections	

### Refinement

**Table:**

Refinement on *F*^2^	Primary atom site location: structure-invariant direct methods
Least-squares matrix: full	Secondary atom site location: difference Fourier map
*R*[*F*^2^ > 2σ(*F*^2^)] = 0.047	Hydrogen site location: inferred from neighbouring sites
*wR*(*F*^2^) = 0.105	H atoms treated by a mixture of independent and constrained refinement
*S* = 1.01	*w* = 1/[σ^2^(*F*_o_^2^) + (0.0373*P*)^2^] where *P* = (*F*_o_^2^ + 2*F*_c_^2^)/3
4041 reflections	(Δ/σ)_max_ = 0.001
245 parameters	Δρ_max_ = 0.58 e Å^−^^3^
2 restraints	Δρ_min_ = −0.47 e Å^−^^3^

### Special details

**Table:**

Geometry. All s.u.'s (except the s.u. in the dihedral angle between two l.s. planes) are estimated using the full covariance matrix. The cell s.u.'s are taken into account individually in the estimation of s.u.'s in distances, angles and torsion angles; correlations between s.u.'s in cell parameters are only used when they are defined by crystal symmetry. An approximate (isotropic) treatment of cell s.u.'s is used for estimating s.u.'s involving l.s. planes.

### Fractional atomic coordinates and isotropic or equivalent isotropic displacement parameters (Å^2^)

**Table:**

	*x*	*y*	*z*	*U*_iso_*/*U*_eq_	
Br1	−0.18793 (4)	0.67333 (3)	0.911231 (19)	0.01841 (12)	
O1	0.4430 (3)	0.3220 (2)	0.53800 (14)	0.0156 (5)	
H1	0.409 (7)	0.351 (5)	0.575 (2)	0.11 (2)*	
O2	0.3101 (2)	0.44759 (19)	0.61666 (13)	0.0160 (5)	
N1	0.3917 (3)	0.3741 (2)	0.40520 (15)	0.0126 (6)	
N2	0.3061 (3)	0.4591 (2)	0.35913 (16)	0.0148 (6)	
N3	0.1528 (3)	0.5638 (2)	0.70229 (17)	0.0160 (6)	
H3	0.218 (3)	0.510 (2)	0.694 (2)	0.034 (12)*	
C1	0.4707 (3)	0.2917 (3)	0.36862 (19)	0.0142 (7)	
C2	0.4743 (4)	0.3022 (3)	0.2904 (2)	0.0174 (7)	
H2	0.4249	0.3651	0.2617	0.021*	
C3	0.5495 (4)	0.2218 (3)	0.2542 (2)	0.0205 (8)	
H3A	0.5514	0.2293	0.2006	0.025*	
C4	0.6222 (4)	0.1298 (3)	0.2955 (2)	0.0190 (8)	
H4	0.6733	0.0740	0.2705	0.023*	
C5	0.6196 (4)	0.1204 (3)	0.3735 (2)	0.0197 (8)	
H5	0.6701	0.0578	0.4019	0.024*	
C6	0.5448 (4)	0.2005 (3)	0.4112 (2)	0.0176 (7)	
H6	0.5442	0.1932	0.4649	0.021*	
C7	0.3772 (3)	0.3860 (3)	0.48029 (19)	0.0131 (7)	
C8	0.2809 (3)	0.4804 (3)	0.48482 (19)	0.0129 (7)	
C9	0.2418 (3)	0.5222 (3)	0.40686 (18)	0.0130 (7)	
C10	0.1464 (4)	0.6231 (3)	0.37541 (19)	0.0184 (7)	
H10A	0.1370	0.6263	0.3190	0.028*	
H10B	0.1978	0.6922	0.3989	0.028*	
H10C	0.0418	0.6172	0.3877	0.028*	
C11	0.2435 (3)	0.5109 (3)	0.55732 (19)	0.0155 (7)	
C12	0.1439 (3)	0.6012 (3)	0.56902 (19)	0.0154 (7)	
H12	0.1024	0.6472	0.5254	0.018*	
C13	0.1018 (3)	0.6283 (3)	0.63815 (19)	0.0152 (7)	
C14	0.0010 (4)	0.7306 (3)	0.6434 (2)	0.0178 (7)	
H14A	0.0427	0.7721	0.6914	0.027*	
H14B	−0.1065	0.7062	0.6435	0.027*	
H14C	0.0014	0.7801	0.5987	0.027*	
C15	0.1320 (4)	0.5704 (3)	0.77953 (19)	0.0165 (7)	
C16	0.2537 (4)	0.5276 (3)	0.8356 (2)	0.0242 (8)	
H16	0.3453	0.4987	0.8208	0.029*	
C17	0.2420 (4)	0.5269 (3)	0.9128 (2)	0.0256 (9)	
H17	0.3254	0.4963	0.9504	0.031*	
C18	0.1116 (4)	0.5696 (3)	0.9364 (2)	0.0220 (8)	
H18	0.1046	0.5707	0.9896	0.026*	
C19	−0.0089 (4)	0.6110 (3)	0.87941 (19)	0.0154 (7)	
C20	−0.0032 (4)	0.6109 (3)	0.80153 (19)	0.0172 (7)	
H20	−0.0893	0.6377	0.7639	0.021*	

### Atomic displacement parameters (Å^2^)

**Table:**

	*U*^11^	*U*^22^	*U*^33^	*U*^12^	*U*^13^	*U*^23^
Br1	0.01953 (19)	0.0200 (2)	0.01736 (19)	0.00189 (14)	0.00775 (13)	−0.00017 (14)
O1	0.0169 (11)	0.0183 (13)	0.0119 (12)	0.0042 (10)	0.0035 (9)	0.0031 (10)
O2	0.0196 (11)	0.0165 (12)	0.0120 (12)	0.0033 (10)	0.0034 (9)	−0.0002 (10)
N1	0.0147 (13)	0.0130 (14)	0.0110 (14)	0.0010 (11)	0.0043 (10)	0.0009 (11)
N2	0.0159 (13)	0.0132 (14)	0.0150 (15)	0.0020 (12)	0.0024 (11)	0.0021 (12)
N3	0.0175 (14)	0.0164 (16)	0.0154 (15)	0.0035 (13)	0.0069 (11)	0.0011 (13)
C1	0.0119 (15)	0.0175 (17)	0.0134 (17)	−0.0035 (14)	0.0028 (12)	−0.0040 (14)
C2	0.0162 (16)	0.0213 (19)	0.0148 (18)	−0.0002 (15)	0.0032 (13)	−0.0013 (15)
C3	0.0192 (17)	0.027 (2)	0.0165 (19)	−0.0036 (16)	0.0072 (14)	−0.0036 (16)
C4	0.0150 (16)	0.0172 (18)	0.026 (2)	−0.0022 (15)	0.0072 (14)	−0.0088 (15)
C5	0.0193 (17)	0.0179 (19)	0.022 (2)	0.0018 (15)	0.0036 (14)	−0.0020 (16)
C6	0.0152 (16)	0.0216 (19)	0.0167 (18)	−0.0043 (15)	0.0046 (13)	−0.0020 (15)
C7	0.0141 (15)	0.0160 (17)	0.0097 (16)	−0.0028 (14)	0.0034 (12)	0.0004 (14)
C8	0.0117 (15)	0.0145 (17)	0.0121 (17)	−0.0026 (14)	0.0016 (12)	0.0014 (14)
C9	0.0140 (15)	0.0129 (17)	0.0123 (17)	−0.0014 (14)	0.0031 (12)	0.0016 (14)
C10	0.0221 (17)	0.0194 (19)	0.0146 (18)	0.0035 (15)	0.0055 (14)	−0.0007 (15)
C11	0.0127 (15)	0.0188 (18)	0.0150 (18)	−0.0068 (14)	0.0031 (13)	0.0007 (14)
C12	0.0147 (15)	0.0198 (18)	0.0117 (17)	−0.0013 (14)	0.0028 (12)	0.0017 (14)
C13	0.0129 (15)	0.0164 (18)	0.0163 (18)	−0.0049 (14)	0.0026 (13)	0.0000 (14)
C14	0.0201 (17)	0.0186 (19)	0.0151 (18)	−0.0006 (15)	0.0043 (13)	−0.0016 (14)
C15	0.0205 (16)	0.0162 (18)	0.0136 (18)	−0.0024 (15)	0.0051 (13)	−0.0002 (14)
C16	0.0202 (17)	0.033 (2)	0.020 (2)	0.0076 (17)	0.0049 (14)	0.0007 (17)
C17	0.0235 (18)	0.036 (2)	0.0172 (19)	0.0091 (17)	0.0031 (14)	0.0048 (17)
C18	0.0273 (18)	0.026 (2)	0.0129 (18)	−0.0004 (16)	0.0039 (14)	0.0031 (15)
C19	0.0177 (16)	0.0133 (18)	0.0174 (18)	0.0001 (14)	0.0090 (13)	0.0004 (14)
C20	0.0165 (16)	0.0220 (19)	0.0129 (17)	−0.0016 (15)	0.0025 (13)	−0.0009 (15)

### Geometric parameters (Å, º)

**Table:**

Br1—C19	1.906 (3)	C8—C11	1.425 (4)
O1—C7	1.303 (4)	C8—C9	1.433 (4)
O1—H1	0.843 (10)	C9—C10	1.493 (4)
O2—C11	1.320 (4)	C10—H10A	0.9800
N1—C7	1.359 (4)	C10—H10B	0.9800
N1—N2	1.406 (4)	C10—H10C	0.9800
N1—C1	1.417 (4)	C11—C12	1.415 (5)
N2—C9	1.327 (4)	C12—C13	1.376 (4)
N3—C13	1.360 (4)	C12—H12	0.9500
N3—C15	1.409 (4)	C13—C14	1.506 (5)
N3—H3	0.881 (10)	C14—H14A	0.9800
C1—C2	1.389 (4)	C14—H14B	0.9800
C1—C6	1.395 (5)	C14—H14C	0.9800
C2—C3	1.379 (5)	C15—C16	1.391 (4)
C2—H2	0.9500	C15—C20	1.395 (5)
C3—C4	1.386 (5)	C16—C17	1.382 (5)
C3—H3A	0.9500	C16—H16	0.9500
C4—C5	1.381 (5)	C17—C18	1.380 (5)
C4—H4	0.9500	C17—H17	0.9500
C5—C6	1.389 (5)	C18—C19	1.387 (4)
C5—H5	0.9500	C18—H18	0.9500
C6—H6	0.9500	C19—C20	1.381 (4)
C7—C8	1.405 (4)	C20—H20	0.9500
			
C7—O1—H1	102 (4)	H10A—C10—H10B	109.5
C7—N1—N2	110.2 (3)	C9—C10—H10C	109.5
C7—N1—C1	131.3 (3)	H10A—C10—H10C	109.5
N2—N1—C1	118.5 (3)	H10B—C10—H10C	109.5
C9—N2—N1	106.1 (3)	O2—C11—C12	119.7 (3)
C13—N3—C15	133.3 (3)	O2—C11—C8	115.0 (3)
C13—N3—H3	113 (3)	C12—C11—C8	125.3 (3)
C15—N3—H3	114 (3)	C13—C12—C11	125.6 (3)
C2—C1—C6	120.1 (3)	C13—C12—H12	117.2
C2—C1—N1	119.9 (3)	C11—C12—H12	117.2
C6—C1—N1	120.0 (3)	N3—C13—C12	120.2 (3)
C3—C2—C1	120.2 (3)	N3—C13—C14	119.7 (3)
C3—C2—H2	119.9	C12—C13—C14	120.1 (3)
C1—C2—H2	119.9	C13—C14—H14A	109.5
C2—C3—C4	120.4 (3)	C13—C14—H14B	109.5
C2—C3—H3A	119.8	H14A—C14—H14B	109.5
C4—C3—H3A	119.8	C13—C14—H14C	109.5
C5—C4—C3	119.2 (3)	H14A—C14—H14C	109.5
C5—C4—H4	120.4	H14B—C14—H14C	109.5
C3—C4—H4	120.4	C16—C15—C20	119.5 (3)
C4—C5—C6	121.6 (3)	C16—C15—N3	115.9 (3)
C4—C5—H5	119.2	C20—C15—N3	124.4 (3)
C6—C5—H5	119.2	C17—C16—C15	120.2 (3)
C5—C6—C1	118.6 (3)	C17—C16—H16	119.9
C5—C6—H6	120.7	C15—C16—H16	119.9
C1—C6—H6	120.7	C18—C17—C16	121.4 (3)
O1—C7—N1	125.9 (3)	C18—C17—H17	119.3
O1—C7—C8	126.1 (3)	C16—C17—H17	119.3
N1—C7—C8	108.0 (3)	C17—C18—C19	117.4 (3)
C7—C8—C11	119.8 (3)	C17—C18—H18	121.3
C7—C8—C9	104.4 (3)	C19—C18—H18	121.3
C11—C8—C9	135.8 (3)	C20—C19—C18	123.0 (3)
N2—C9—C8	111.3 (3)	C20—C19—Br1	119.0 (2)
N2—C9—C10	119.1 (3)	C18—C19—Br1	117.9 (3)
C8—C9—C10	129.6 (3)	C19—C20—C15	118.4 (3)
C9—C10—H10A	109.5	C19—C20—H20	120.8
C9—C10—H10B	109.5	C15—C20—H20	120.8
			
C7—N1—N2—C9	0.6 (3)	C11—C8—C9—N2	−178.1 (3)
C1—N1—N2—C9	178.5 (3)	C7—C8—C9—C10	−178.0 (3)
C7—N1—C1—C2	−176.6 (3)	C11—C8—C9—C10	3.2 (6)
N2—N1—C1—C2	5.9 (4)	C7—C8—C11—O2	2.5 (4)
C7—N1—C1—C6	3.2 (5)	C9—C8—C11—O2	−178.9 (3)
N2—N1—C1—C6	−174.2 (3)	C7—C8—C11—C12	−177.7 (3)
C6—C1—C2—C3	0.9 (5)	C9—C8—C11—C12	1.0 (6)
N1—C1—C2—C3	−179.3 (3)	O2—C11—C12—C13	−3.1 (5)
C1—C2—C3—C4	−0.1 (5)	C8—C11—C12—C13	177.0 (3)
C2—C3—C4—C5	−0.5 (5)	C15—N3—C13—C12	179.1 (3)
C3—C4—C5—C6	0.5 (5)	C15—N3—C13—C14	0.0 (5)
C4—C5—C6—C1	0.3 (5)	C11—C12—C13—N3	−2.1 (5)
C2—C1—C6—C5	−0.9 (5)	C11—C12—C13—C14	176.9 (3)
N1—C1—C6—C5	179.2 (3)	C13—N3—C15—C16	−150.3 (3)
N2—N1—C7—O1	−179.2 (3)	C13—N3—C15—C20	33.1 (5)
C1—N1—C7—O1	3.2 (5)	C20—C15—C16—C17	−1.1 (5)
N2—N1—C7—C8	−0.2 (3)	N3—C15—C16—C17	−177.9 (3)
C1—N1—C7—C8	−177.8 (3)	C15—C16—C17—C18	−0.9 (6)
O1—C7—C8—C11	−2.2 (5)	C16—C17—C18—C19	1.4 (6)
N1—C7—C8—C11	178.7 (3)	C17—C18—C19—C20	0.1 (5)
O1—C7—C8—C9	178.8 (3)	C17—C18—C19—Br1	−178.7 (3)
N1—C7—C8—C9	−0.3 (3)	C18—C19—C20—C15	−2.0 (5)
N1—N2—C9—C8	−0.8 (3)	Br1—C19—C20—C15	176.7 (2)
N1—N2—C9—C10	178.1 (3)	C16—C15—C20—C19	2.5 (5)
C7—C8—C9—N2	0.7 (3)	N3—C15—C20—C19	179.0 (3)

### Hydrogen-bond geometry (Å, º)

Cg1 is the centroid of the N1/N2/C7–C9 ring.

**Table:**

*D*—H···*A*	*D*—H	H···*A*	*D*···*A*	*D*—H···*A*
O1—H1···O2	0.84 (1)	1.68 (3)	2.468 (3)	154 (6)
N3—H3···O2	0.88 (1)	1.87 (3)	2.617 (4)	142 (4)
C14—H14*B*···*Cg*1^i^	0.98	2.69	3.495 (4)	140

Symmetry code: (i) −*x*, −*y*+1, −*z*+1.
